# Development and validation of a diagnostic nomogram to evaluate tubular atrophy/interstitial fibrosis of IgA nephropathy

**DOI:** 10.7150/ijms.91804

**Published:** 2024-02-04

**Authors:** Yangang Gan, Yihuan Cai, Jiajia Li, Jianping Wu, Rui Zhang, Qianqian Han, Wenchao Li, Qiongqiong Yang

**Affiliations:** Department of Nephrology, Sun Yat-sen Memorial Hospital, Sun Yat-sen University, Guangzhou, China.

**Keywords:** estimated glomerular filtration rate, IgA nephropathy, nomogram, pathological change

## Abstract

**Background:** IgA nephropathy (IgAN) is a cause of chronic kidney disease (CKD). Tubular atrophy/interstitial fibrosis is associated with IgAN prognosis. However, simple tools for predicting pathological lesions of IgAN remain limited. Our objective was to develop a tool for evaluating tubular atrophy/interstitial fibrosis in patients with IgAN.

**Methods:** In this cross-sectional study, 410 biopsy-verified IgAN patients were included. The factors associated with the incident interstitial fibrosis or tubular atrophy in IgAN were confirmed by using logistic regression analysis. A nomogram was developed using logistic regression coefficients to evaluate tubular atrophy or interstitial fibrosis. Receiver operating characteristic curves (ROC) and calibration curves were used to determine the discriminative ability and predictive accuracy of the nomogram.

**Results:** In this study, the IgAN patients with tubular atrophy or interstitial fibrosis were older and had a higher percentage of males, hypertension and urinary protein excretion (UPE), with high levels of serum cystatin C, serum creatinine, high-sensitivity C-reactive protein and serum C4. The eGFRcr-cys equation calculated using serum creatinine, cystatin C and UPE were considered independent influencing factors of tubular atrophy or interstitial fibrosis in patients with IgAN. Furthermore, the nomogram demonstrated good discrimination (AUC: 0.87, 95% CI 0.81 to 0.93) and calibration in the validation cohort.

**Conclusion:** The eGFRcr-cys and UPE are associated with tubular atrophy or interstitial fibrosis in patients with IgAN. Diagnostic nomogram can predict tubular atrophy or interstitial fibrosis in IgAN.

## Introduction

Immunoglobulin A nephropathy (IgAN) is a type of glomerulonephritis with a high incidence in the world 1. IgA deposition in glomeruli is the main characteristic of IgAN 2. IgA is commonly deposited in the mesangial region of the glomerulus, which induces an inflammatory response that promotes mesangial proliferation and interstitial damage that progresses to end-stage renal disease (ESRD) in nearly 40% of IgAN patients 3. Patients with IgAN have many different clinical symptoms, ranging from asymptomatic hematuria and proteinuria to massive proteinuria to acute renal failure 4. Currently, renal biopsy is crucial for the diagnosis and prognosis of IgAN. The Oxford classification is also used in clinical practice to assess the condition of patients with IgAN. Tubular atrophy or interstitial fibrosis lesions are vital indicators of the outcome of IgAN 5, 6. Kidney puncture is an invasive examination that is difficult to repeat frequently. Simple tools for predicting tubular atrophy or interstitial fibrosis in IgAN are limited.

Estimated glomerular filtration rate (eGFR) is also an indicator of renal function 7. There was evidence that the equation of eGFR incorporating cystatin C will improve the accuracy of predicting outcomes such as death and ESRD 8, 9. Cystatin C can also elevate the accuracy of eGFR especially in patients with muscle wasting or chronic illness 10. The eGFRcr-cys, which uses both markers, including creatinine and cystatin C, has higher accuracy than either eGFRcr or eGFRcys alone 11. In this process, eGFR is a method frequently used to evaluate the renal function of IgAN, but its value in evaluating pathological lesions was unclear. Therefore, we aimed to explore the value of eGFRcr-cys in IgAN and establish a diagnostic nomogram to predict tubular atrophy or interstitial fibrosis in patients with IgAN.

## Methods

### Study population and design

This was a cross-sectional study. Patients diagnosed with IgAN by renal biopsy at Sun Yat-sen Memorial Hospital from 2015 to 2023 were included in the study. Patients with secondary IgAN, such as systemic lupus erythematosus, Henoch-Schonlein purpura nephritis, hepatitis B virus-related glomerulonephritis, diabetic nephropathy, and missing data were excluded. Ultimately, 410 patients with IgAN were included and randomly divided into derivation and validation cohorts at a ratio of 7:3.

### Clinical and laboratory data

The clinical and laboratory information of the patients included in this study was collected. The clinical indicators included sex, age, height, weight, blood pressure, and disease course. Laboratory data included hemoglobin, serum creatinine, serum cystatin C, serum albumin, high-sensitivity C-reactive protein (hs-CRP), serum immunoglobulin, and complement levels. The BMI was calculated as weight (kg)/height (m^2^).

### The equations of eGFR

Creatinine-based eGFR was calculated using the equation described in previous research 7. eGFR was calculated using cystatin C and creatinine according to equations described by the chronic kidney disease epidemiology collaboration 11.

### Renal pathology evaluation

Light microscopy and immunofluorescence were used to test all IgAN specimens. We used the Oxford Classification Scoring System to assess and classify histologic lesions, including mesangial hypercellularity (M), endocapillary hypercellularity (E), segmental glomerulosclerosis (S), interstitial fibrosis/tubular atrophy (T), and cellular/fibrocellular crescents (C) 12. At least two pathologists independently evaluated histopathological manifestations.

### Statistical analysis

Continuous variables were expressed as mean ± SD or median and interquartile range. Categorical variables were expressed as frequencies and percentages. The Χ^2^ test was used to compare differences between categorical variables. Binary logistic regression analysis was used to evaluate factors associated with incident interstitial fibrosis or tubular atrophy in IgAN. Nomogram construction was performed according to logistic regression analysis. Receiver operating characteristic (ROC) and calibration curves were used to determine the discriminative ability and predictive accuracy of the nomogram. Statistical Product and Service Solutions (SPSS) version 25.0, and R 4.0.4, were utilized for statistical analyses.* P*<0.05.

## Results

### Clinicopathological characteristics of the included patients

The present study included 410 patients with IgAN who met the inclusion criteria. The characteristics of the study population are summarized in Table 1. Among the 410 patients, 133 (32.4%) were male and 277 (67.6%) were female, with a median age of 34 years (range 28-45 years). There were no significant differences between the derivation and validation cohorts (all *P* > 0.05).

### Characteristics in patients with tubular atrophy or interstitial fibrosis of IgAN

In the derivation cohort, 34 (11.7%) and 48 (16.6%) patients were assigned to the T2 and T1 groups, respectively. The remaining patients were classified as T0 group. Table 2 presents the results. The T ≥ 1 group was older, had a higher proportion of male and had hypertension, which was also characterized by a significantly lower level of serum albumin, eGFRcr-cys, serum IgG, and serum IgM, with a higher level of urinary protein excretion (UPE), serum creatinine, serum cystatin C, Hs-CRP and serum C4.

### Nomogram development

**Figure 2:**
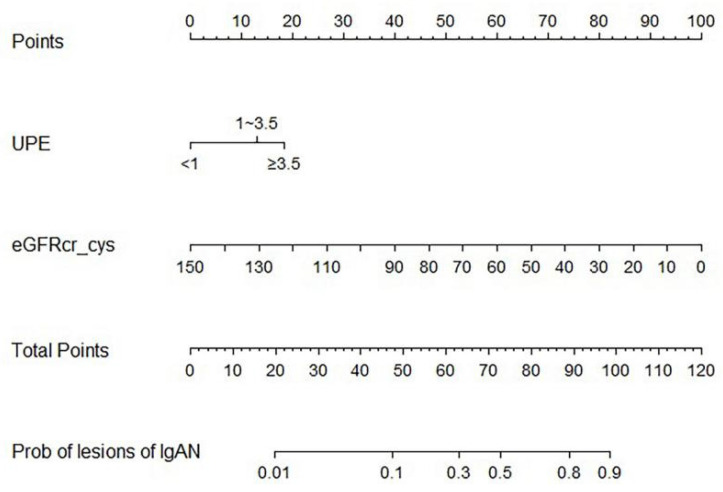
Diagnostic nomogram. To use the nomogram, draw a line perpendicular from the corresponding axis of each risk factor until it reaches the top line labeled “Points”. Sum up the number of points for all risk factors then draw a line descending from the axis labeled “Total points” until it determined the probabilities of tubular atrophy/interstitial fibrosis.

Logistic regression was used to determine the factors influencing tubular atrophy or interstitial fibrosis in IgAN, as shown in Table 3. eGFRcr-cys is composed of cystatin C, creatinine, age, and sex. Finally, hypertension, UPE, serum albumin, serum IgG, serum C4, and eGFRcr-cys were incorporated into the analysis showed in Figure 2. After adjusting for confounding factors, independent influencing factors were eGFRcr-cys and UPE. Based on the results of the multivariate analysis, we built a nomogram for predicting tubular atrophy or interstitial fibrosis of IgAN (Figure 2). First, we project the values of UPE and eGFRcr_cys onto the positions corresponding to the line of “Points” to get the score of each factor, and then sum them to obtain the total points. Based on the total points projection onto the last row, the risk of tubular atrophy/interstitial fibrosis is calculated. Higher total points indicate an elevated risk of tubular atrophy/interstitial fibrosis.

### Nomogram validation

**Figure 3:**
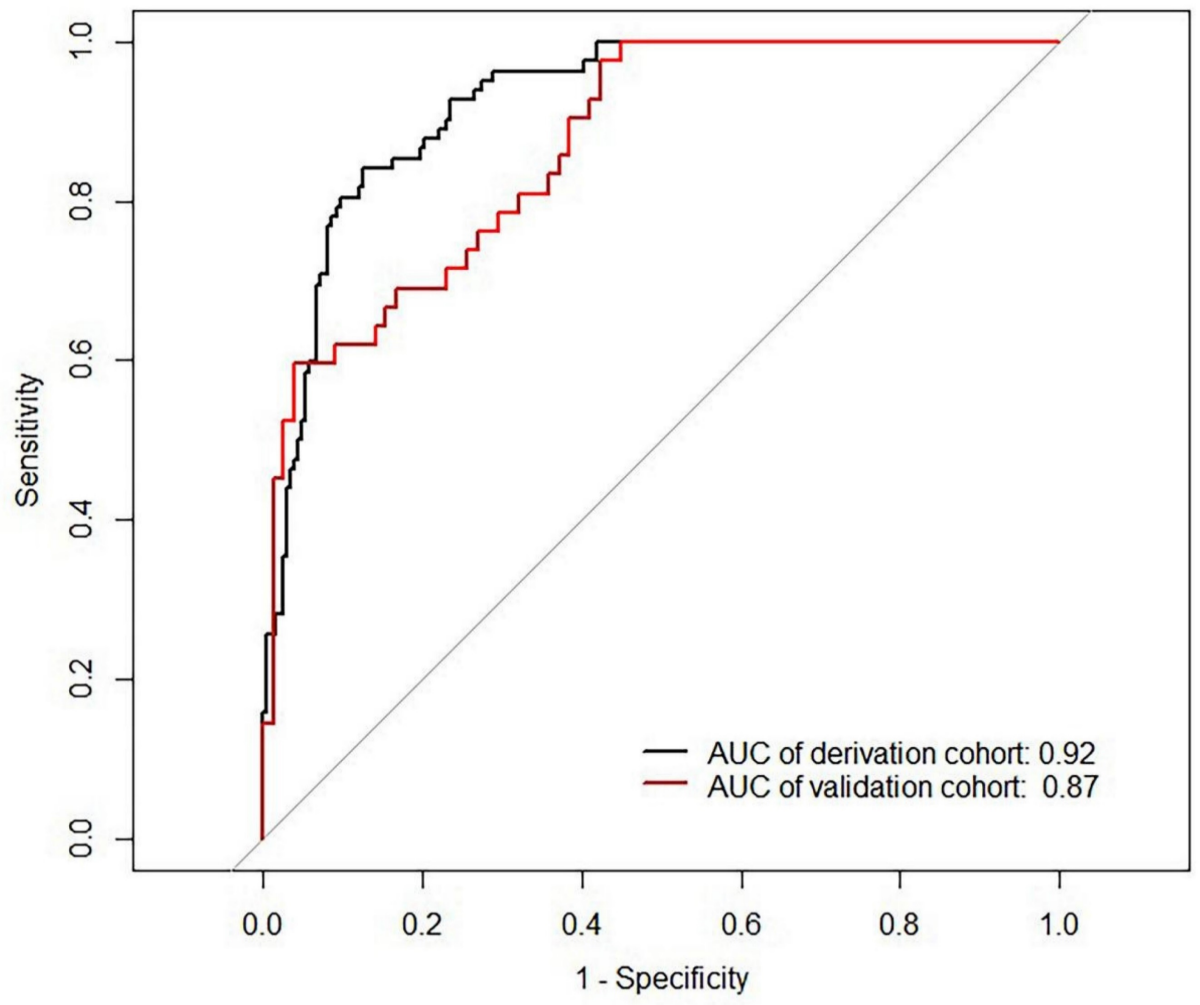
** ROC curves of the nomogram.** AUC: area under the curve.

**Figure 4:**
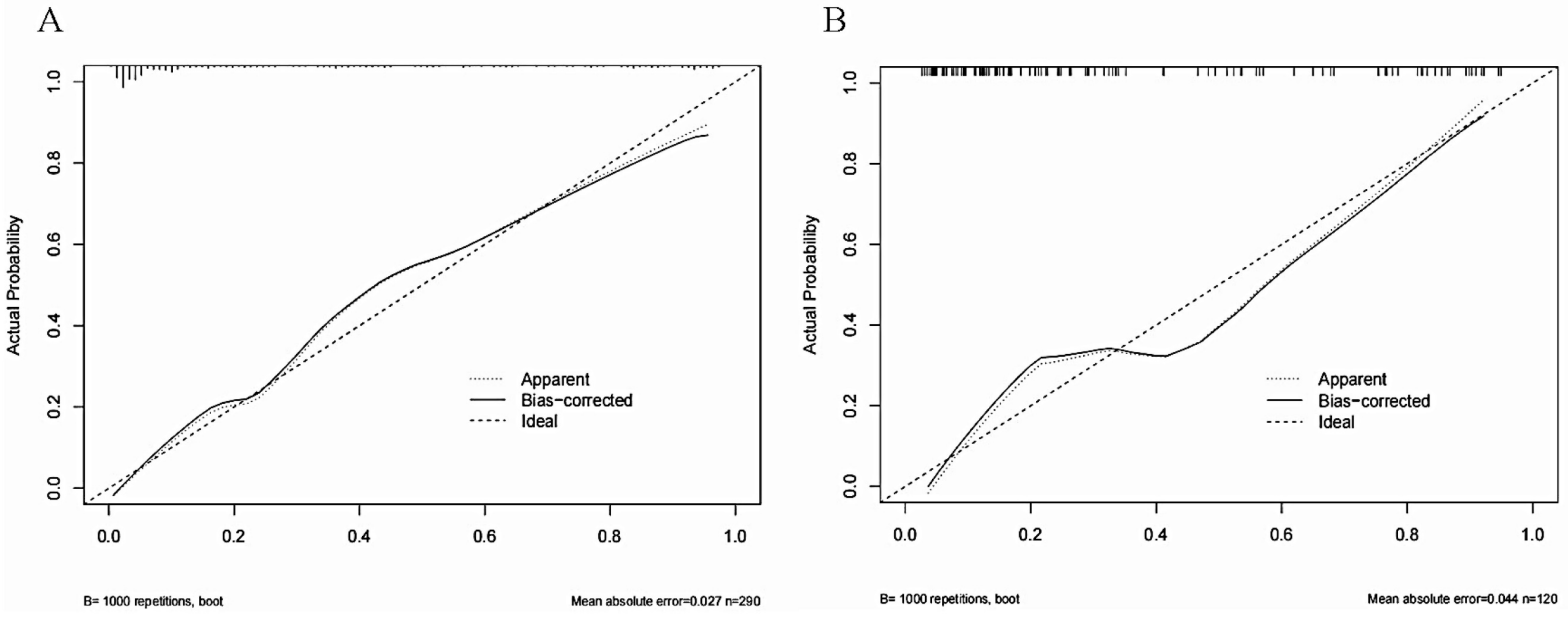
The calibration curves of the nomogram for predicting the T1/2 lesions of IgAN patients at (A) in the derivation cohort and (B) in the validation cohort. The X-axis represents the nomogram-predicted probability of diagnosis, and the Y-axis represents the actual probability estimated with the logistic regression methods.

The Area Under Curve was 0.92[ 95% CI (0.90 - 0.95), *P*< 0.001] in the derivation cohort and 0.87[ 95% CI (0.81 - 0.93), *P* < 0.001] in the validation cohort (Figure 3). The calibration curves also revealed that our nomogram accurately predicted tubular atrophy or interstitial fibrosis of IgAN (Figure 4A, B).

## Discussion

The equation of eGFRcr-cys can estimate GFR more accurately than the equation using cystatin C or creatinine alone, and the differences from measured GFR are smaller 13. In this study, we explored the value of eGFRcr-cys in patients with IgAN for Oxford T lesions. IgAN patients with decreased basal eGFRcr-cys had a high risk of renal fibrosis or tubular atrophy.

IgAN is a series of diseases mainly characterized by immune complex deposition and cell proliferation in the glomerular mesangial area 14. Oxford T lesions, including T0/T1/T2, are important pathological features of IgAN, as evaluated by the Oxford classification. Studies have confirmed that Oxford T lesions are an independent risk factor for IgAN prognosis of IgAN^15^, ^16^. MEST score and clinical data can be used to predict the risk of IgAN patients 17. Currently, Oxford T lesions can be determined through renal biopsy, which is invasive and difficult to repeat in IgAN patients 18. IgAN is a chronic disease with variable clinical outcomes. The international glomerulonephritis guidelines recommend that the management of IgAN should focus on supportive care to slow disease progression of the disease 19. In this process, eGFR is frequently used to evaluate the renal function of IgAN, but its value in evaluating pathological lesions is unclear.

eGFR is a common indicator used to assess kidney function in clinical practice worldwide. A decline in GFR to 60 ml/min/1.73 m^2^ or lower for 3 months or longer is a criterion to diagnose chronic kidney disease (CKD), which is also related to adverse outcomes including death 20, 21. Thomas Knoop.et al found that inclusion of eGFR and age in prognostic models of ESRD and death can improve model accuracy in IgAN patients 22. The eGFRcr equation is commonly used worldwide in clinical practice. However, a larger bias in the eGFRcr equations was observed in GFR estimates for different ethnic groups, which led to differences in the estimates of GFR stage 13. Patient age, sex, and muscle mass may affect serum creatinine values 23. Therefore, the function of the kidney based on serum creatinine level may be overestimated because of poor nutritional status and reduced muscle 24, 25. Thio et al. found that a genetic risk score is strongly related to baseline CKD and eGFR independent of known risk factors, which may suggest the underlying genetics of renal function rather than creatinine metabolism or underlying etiology 26.

Compared to serum creatinine, cystatin C levels are not affected by factors such as sex and muscle mass 27. Studies have found that cystatin C provides more accurate results in patients with an eGFRcr of 60 to 74 ml/minute/1.73 m^2^ without proteinuria, muscle wasting, or chronic disease 10. Fleming et al. also found that serum cystatin C is a more useful biomarker for estimating eGFR in an observational cohort study, especially for patients with baseline eGFR< 60ml/min/1.73m^2^ 28. Studies have found that eGFRcr-cys equations minimize inaccuracy in both ethnic groups, and the use of eGFRcr-cys may enhance the accuracy of CKD diagnosis and GFR staging 13. The new eGFRcr-cys equation excluding race is more accurate in measuring GFR than equations using creatinine or cystatin C levels alone and resulted in lesser differences from measured GFR 11, 13. However, it has not been extensively studied in patients with IgAN. This study found that the equation of eGFRcr-cys is an independent influencing factor for Oxford T lesions in patients with IgAN. Thus, eGFRcr-cys may be a useful marker for predicting Oxford T lesions in IgAN.

Predicting pathological lesions in IgAN patients is challenging because IgAN is highly heterogeneous. Thus, there is an urgent need to develop a simple tool to predict IgAN. In this study, we found that eGFRcr-cys and UPE were independent risk factors for IgAN with T lesions. Proteinuria is a widely studied risk factor for the progression to ESRD in patients with IgAN. Thompson et al. suggested the reduction of proteinuria as a surrogate endpoint in trials of IgAN^29^. We established a diagnostic nomogram based on the results of the logistic regression model. In the validation cohort, the Area Under Curve of the nomogram was 0.87 (95% CI 0.81 - 0.93, *P* < 0.001), indicating that our diagnostic nomogram had great internal validation and performed well in discrimination and calibration. This may provide vital clues for predicting T lesions of IgAN and may be useful in clinical practice.

Our study has some limitations. A causal relationship could not be drawn because of the cross-sectional design. Moreover, our data were all from renal biopsy patients, and some patients initially did not undergo renal biopsy for some reason. Therefore, missing data and selection bias might have been present in our study.

In conclusion, eGFRcr-cys and UPE were associated with tubular atrophy or interstitial fibrosis in patients with IgAN. Diagnostic nomograms can predict tubular atrophy or interstitial fibrosis in patients with IgAN.

## Figures and Tables

**Figure 1:**
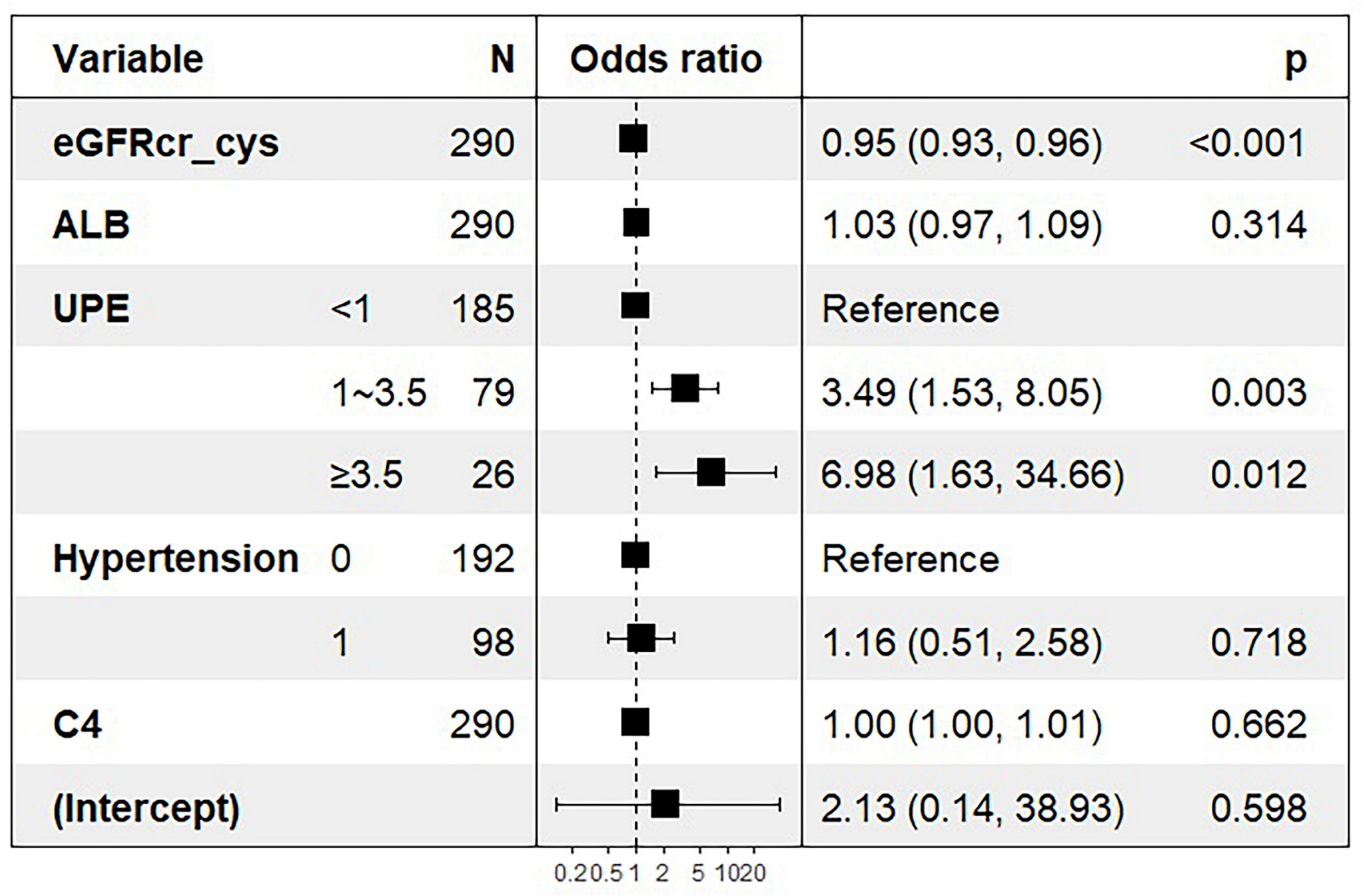
ORs for tubular atrophy/interstitial fibrosis associated with eGFRcr-cys and UPE. ORs are from a multivariable-adjusted model including hypertension, serum albumin, serum IgG, serum C4, eGFRcr-cys and UPE. Boxes in the graph represent the OR and lines/arrows represent the 95% CI.

**Table 1:** Baseline characteristics of the patients in the derivation and validation cohorts

Characteristic	Total (N=410)	Derivation cohort (N=290)	Validation cohort (N=120)	*P*
Age (years)	34(28, 45)	34(28, 44)	34(27, 47)	0.569
Men, n (%)	133(32.44)	93(32.07)	40(33.33)	0.803
BMI (kg/m^2^)	22.50(20.50, 24.96)	22.42(20.70, 25.13)	22.50(20.30, 24.30)	0.552
Hypertension, n (%)	135(32.93)	98(33.79)	37(30.83)	0.562
UPE (g/d)				0.182
<1	261(63.66)	185(63.79)	76(63.33)	
1-3.5	118(28.78)	79(27.24)	39(32.50)	
≥3.5	31(7.56)	26(8.97)	5(4.17)	
Hematuria (RBCs/ul)	54.00(20.00, 149.25)	54.00(18.00, 150.00)	61.00(22.25, 146.25)	0.341
Serum creatinine (μmol/L)	89.00(71.75, 125.00)	89.00(71.00, 125.00)	89.00(73.00, 121.00)	0.632
Serum cystatin C	1.00(0.82, 1.37)	0.97(0.82, 1.36)	1.02(0.84, 1.39)	0.504
Serum albumin (g/L)	36.40(32.70, 40.02)	36.75(32.78, 40.30)	35.60(32.60, 39.48)	0.272
Hs-CRP (mg/L)	0.78(0.32, 1.99)	0.80(0.31, 1.99)	0.66(0.33, 2.01)	0.936
eGFRcr-cys (mL/min/1.73m2)	80.44(50.48, 103.30)	81.27(50.30, 104.00)	78.60(54.69, 101.44)	0.552
Serum IgG (g/L)	11.80(9.55, 13.80)	11.90(9.77, 13.83)	11.60(9.35, 13.48)	0.307
Serum IgA (g/L)	3.16(2.51, 3.99)	3.15(2.49, 3.98)	3.18(2.60, 4.07)	0.744
Serum IgM (g/L)	1.08(0.79, 1.47)	1.08(0.80, 1.43)	1.04(0.76, 1.55)	0.938
Serum C3 (mg/L)	1030.00(916.75, 1170.00)	1040.00(920.00, 1170.00)	1005(895, 1178)	0.785
Serum C4 (mg/L)	246.00(201.00, 305.25)	247.50(203.00, 305.00)	243(198, 308)	0.809
Oxford MEST-C, n (%)				
M1	385(93.90)	270(93.10)	115(95.8)	0.293
E1	81(19.80)	58(20.00)	23(19.2)	0.874
S1	196(47.80)	137(47.20)	59(49.2)	0.723
T1/2	124(30.20)	82(28.30)	42(35.0)	0.177
C1/2	221(53.90)	149(51.40)	72(60.0)	0.111

Results are presented as mean SD, median (interquartile range), or number (percentage). Groups were compared by using Kruskal-Wallis or F test *BMI* body mass index; *UPE* urinary protein excretion; *eGFR* estimated glomerular filtration rate;* hs-CRP* high-sensitivity C-reactive protein; *MEST-C*, *M* mesangial hypercellularity; *E* endocapillary hypercellularity; *S* segmental glomerulosclerosis; *T* interstitial fibrosis/tubular atrophy; *C* crescent formation.

**Table 2:** Baseline pathological characteristics stratified by Oxford T lesions of IgAN patients

Variables	T0 (n 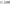 )	T1/2(n 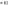 )	*P*
Age (years)	33(27, 42)	37(31, 46)	0.018
Men, n (%)	59(28.37)	34(41.46)	0.023
BMI (kg/m^2^)	22.15(20.53, 24.80)	23.35(21.08, 25.80)	0.072
Hypertension, n (%)	46(22.12)	52(63.41)	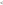 0.001
UPE (g/d)			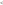 0.001
<1	164(78.84)	21(25.61)	
1-3.5	38(18.27)	41(50.00)	
≥3.5	6(2.88)	20(24.39)	
Hematuria (RBCs/ul)	58.00(19.00,176.50)	45.00(14.75,104.00)	0.205
Serum creatinine (μmol/L)	78.00(66.00, 96.00)	17.50(117.75, 229.75)	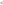 0.001
Serum albumin (g/L)	37.50(34.20, 40.55)	34.60(30.28, 38.80)	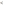 0.001
Serum cystatin C	0.88(0.75, 1.07)	1.61(1.28, 2.43)	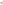 0.001
Hs-CRP (mg/L)	0.69(0.30, 1.74)	0.96(0.34, 3.06)	0.094
eGFRcr-cys (mL/min/1.73m^2^)	95.39(77.55, 108.14)	40.21(22.08, 61.42)	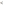 0.001
Serum IgG (g/L)	12.15(10.23, 13.88)	10.85(8.48, 13.83)	0.008
Serum IgA (g/L)	3.25(2.51, 3.98)	2.89(2.46, 4.03)	0.296
Serum IgM (g/L)	1.12(0.82, 1.49)	0.97(0.71, 1.37)	0.049
Serum C3 (mg/L)	1040.00(929.25, 1170.00)	1030.00(916.25, 1142.50)	0.677
Serum C4 (mg/L)	237.00(198.00, 289.00)	278.00(222.75, 336.75)	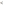 0.001

Results are presented as mean SD, median (interquartile range), or number (percentage). Groups were compared by using Kruskal-Wallis or F test *BMI* body mass index; *UPE* urinary protein excretion; *eGFR* estimated glomerular filtration rate; *hs-CRP* high-sensitivity C-reactive protein; *MEST-C*, *M* mesangial hypercellularity; *E* endocapillary hypercellularity; *S* segmental glomerulosclerosis; *T* interstitial fibrosis/tubular atrophy; *C* crescent formation.

**Table 3:** Analysis of influencing factors about Oxford T lesions in derivation cohort

Variables	Univariate			Multivariate		
	OR	95% CI	*P*	OR	95% CI	*P*
Age (years)	1.02	(1.00-1.04)	0.05			
Gender (Men/Women)	0.56	(0.33, 0.96)	0.03			
Hypertension	6.10	(3.53-10.76)	<0.001	1.16	(0.51-2.58)	0.72
UPE(g/d)			<0.001			
<1	1			1		
1-3.5	8.43	(4.53, 16.14)	<0.001	3.49	(1.53 -8.05)	0.003
≥3.5	26.03	(9.91, 78.17)	<0.001	6.98	(1.63 - 34.66)	0.01
Serum creatinine	1.03	(1.02, 1.04)	<0.001			
Serum albumin	0.94	(0.90, 0.97)	0.001	1.03	(0.97-1.09)	0.31
Serum cystatin C	13.52	(6.81, 30.15)	<0.001			
Hs-CRP	1.00	(0.98, 1.02)	0.820			
eGFRcr-cys	0.94	(0.92, 0.95)	<0.001	0.95	(0.93-0.96)	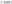
Serum IgG	0.93	(0.86, 1.01)	0.085			
Serum IgM	0.70	(0.40, 1.17)	0.181			
Serum C4	1.00	(1.00, 1.01)	0.001	1.00	(0.97- 1.01)	0.66

*UPE* urinary protein excretion;* hs-CRP* high-sensitivity C-reactive protein;* eGFR* estimated glomerular filtration rate
